# Supplementary material for: Inter-Individual Variability in tDCS Effects: A Narrative Review on the Contribution of Stable, Variable, and Contextual Factors
Source: Brain Sci. 2022 Apr 20;12(5):522. doi: 10.3390/brainsci12050522 (PMC9139102; doi:10.3390/brainsci12050522)
Supplement: Supplementary file 1 [file brainsci-12-00522-s001.zip › brainsci-1625857-supplementary.pdf]

Supplementary Materials

Table S1 shows studies investigating the prevalence of responders and non-responders in their samples.

Study	N	Protocol	Electrodes position	Stimulation type	Measures	TMS protocols	Responders' determination	Responders' %	Results
Ammann et al., 2017 [29]	12	7 minutes; 25 cm^2; 2 or 1 mA	target: left M1 (FDI hotspot); ref: right supraorbital	atDCS, sham (w)	MEPs (pre-tDCS, T0, T15, T30)	1 mV baseline; 10 MEPs per session; pulses every 7+-1 s; right FDI	(i) grand average: CSE > 1.3; (ii) sham-SD based threshold	2 mA condition: 52.8%; 1 mA condition: 33.3%; sham 13.9%	At the group level, 2mA atDCS increased MEPs in all post-stimulation conditions, while 1 mA tDCS significantly increased MEPs only in the post30 condition compared to the pre-tDCS session.
Chew et al., 2015 [30]	29	10 minutes; target: 4x4 cm, ref: 5x7; 0.2, 0.5[a], 1, 2 mA (w)	target: left M1 (hotspot); ref: right supraorbital	atDCS	MEPs (pre-tDCS, T0, T10, T20, T30)	1 mV or 130% rMT (the lowest intensity); 20 MEPs per session; 0.25 Hz; right FDI	(i) grand average: CSE > 1.2; (ii) two steps cluster analysis	72% (responders to at least 1 stimulation intensity condition)	No effects at the group level. Cluster analysis identified two clusters at 0.2 and 2 mA. Considering **only** responders: 67% responded to one intensity, 19% to two intensities, 14% to four intensities.
Labruna et al., 2016 [31]	34[b]	atDCS 13 minutes, ctDCS 9 minutes; 7x5 cm; 1 mA	target: left M1 (ADM hotspot); ref: right supraorbital	atDCS, ctDCS (w)	MEPs (pre-tDCS, every 5 minutes from T0 to T30, T60, T90, T120)	1 mV baseline; 25 MEPs per session; 0.25 Hz; right ADM	grand average: normalized MEPs >1 increase, <1 reduction	atDCS 92% - ctDCS NR	At the group level, atDCS increased MEPs, and ctDCS decreased them. Moreover, atDCS induced larger MEPs in individuals more sensitive to TMS. No correlation was found for ctDCS.

López-Alonso et al., 2014 [26]	56	13 minutes; 7 x 5; 1 mA	target: left M1 (FDI hotspot); ref: right supraorbital	atDCS	MEPs, SICI (pre-tDCS, every five minutes from T0 to T60).	1 mV baseline; 20 MEPs at baseline, 12 MEPs per session; right FDI	(i) grand average: CSE >1; (ii) two-steps cluster analysis	45% according to grand average categorization; 50% according to the cluster analysis.	No effects at the group level. Responders' subgroup showed MEPs increase from 10 minutes after the end of the stimulation compared to the baseline.
López-Alonso et al., 2015 [32]	45	10 minutes; 7x 5 cm; 1 mA	target: left M1(FDI hotspot); ref: right supraorbital	atDCS – (2 sessions 6-12 months apart)	MEPs, SICI (pre-tDCS, every 5 minutes from T0 to T60)	1 mV baseline; MEPs: 20 pulses at baseline, 12 per session; SICI: 20 paired pulses at baseline, 10 per session; right FDI.	grand average: CSE >1	60-64% of responders in each session.	At the group level, atDCS increased excitability after stimulation. 78% of responders in the first session had the same response pattern in the second session.
Luque-Casado et al., 2019 [37]	30	15 minutes; 35 cm^2; 1.5 mA	target: lDLPFC (5 cm anterior to M1-Hand); ref: right supraorbital	atDCS, sham (w)	DSB span (pre-tDCS, T0, T10)	-	hierarchical cluster analysis; k-means cluster analysis	42 - 46%	No effects at the group level. Responders' subset: span score increasing after atDCS compared to T0.
Luque-Casado et al., 2020 [38]	29	15 minutes; 35 cm^2; 1.5 mA	target: lDLPFC (F3); ref: right supraorbital	atDCS, sham (w)	ACC and RTs at n-back and Sternberg tasks (pre-tDCS,	-	hierarchical cluster analysis; k-means cluster analysis	15-59%	No effects at the group level. Responders' subset: improvement after atDCS compared to T0.

					T0, T5)				
Puri et al., 2015 [33]	50[c]	10 - 20 minutes (w); target: 5 × 5 cm, ref: 6 × 8.5 cm; 1.5 mA	target: left M1(FDI hotspot); ref: right supraorbital	atDCS	MEPs (pre-tDCS, every 5 minutes from T0 ti T30)	2 TMS intensities: 130 and 150% of rMT; 15 MEPs per intensity in each session (2 baselines); right FDI.	(i) MEPs normalization to baseline >1; (ii) two-step cluster analysis	46% increased excitability for both atDCS durations; 34% increased at one duration; 20% CSE suppression following both stimulations.	Met carriers showed larger MEPs increasing after 20 minutes atDCS compared to the Val/Val homozygotes group and the 10 minutes condition. A more significant proportion of MET carriers was classified as responders.
Puri et al., 2016 [34]	33[d]	10 - 20 minutes (w); target: 5 cm × 5 cm, ref: 6cm × 8.5 cm; 1.5 mA	target: left M1(FDI hotspot); ref: right supraorbital	atDCS	MEPs (pre-tDCS, every 5 minutes from T0 to T30)	2 TMS intensities: 130 and 150% of rMT; 15 MEPs per intensity in each session (2 baselines); right FDI.	i) grand average > 1.1; (ii) two-step cluster analysis	55% atDCS - 10 minutes - 52% atDCS -20 minutes	At the group level, atDCS increased MEPs compared to T0. Responders' subgroup: 20 minutes of atDCS induced larger MEPs than the 10 minutes condition, especially at late time points (T25 and T30), in which 10-minutes atDCS did not induce MEPs larger than from baseline.
Strube et al., 2015 [35]	29	13 minutes; 7 x 5; 1 mA	target: left M1 (FDI hotspot); ref: right supraorbital	atDCS	MEPs, SICI, ICF, IO-curve (pre-tDCS, every 5 minutes from T0 to T30)	MEPs: 1 mV baseline; 40 MEPs at baseline and 20 MEPs per session; 0.2 Hz; right FDI. IO-curve: increasing intensity 90%, 110%, 130% rMT	grand average: three different cuf offs: > 100%, > 110%, > 150% compared to the baseline	MEPs: > 100% (baseline) = 66%; > 110% = 55%; > 150% = 21%	At the group level, atDCS increased MEPs compared to baseline. Moreover, ICF positively correlated with MEPs amplitude increase. Responders' subgroup: atDCS had higher ICF at baseline compared to non-responders.

						- 7 single pulses per intensity. SICI and ICF: PAS standard protocol: conditioning stimulus 80% rMT and target 1mV.			
Strube et al., 2016 [36]	59	atDCS 13 minutes, ctDCS 9 minutes; 7x5 cm; 1 mA	target: left M1 (hotspot); ref: right supraorbital	atDCS, ctDCS (w)	MEPs (pre-tDCS, T0, T5, T10, T20, T30, T40)	1 mV baseline; 40 MEPs at baseline and 20 MEPs per session; 0.2 Hz; right FDI	(i) grand average: CSE >1 increase, <1 reduction; (ii) hierarchical cluster analysis	atDCS 61%, ctDCS 53%	At the group level, atDCS increased MEPs for 40 minutes, while cTDCS did not affect CSE. Hierarchical cluster analysis detected two clusters within each tDCS condition. In atDCS, cluster 1 showed constant MEPs increase followed by a decrease around 30 - 40 minutes post-stimulation. Cluster 2 revealed MEPs' increasing until 10 minutes after the stimulation, and then MEPs remained stable. CtDCS clusters 1 showed a decrease of CSE stable for the whole post-tDCS period, while cluster 2 revealed an increase of MEPs magnitude.
Tremblay et al., 2016 [39]	40	10 or 20 minutes (b); 7x5 cm; 1 or 2 mA (w)	target: left M1 (hotspot); ref: right supraorbital	atDCS	MEPs (pre-tDCS, T0, T5, T10, T15, T20)	1 mV baseline; 20 MEPs per session; right FDI	grand average, change in CSE when reaching the 95% of the baseline SEM confidence interval: CSE > 1.27	20-35%	No effect at the group level. 10-min atDCS increased CSE for responders' cluster (compared to non-responders) at all post-tDCS recordings for the 2 mA intensity and 1 mA (except T0). 20-min atDCS increased CSE for all recordings after 2 mA tDCS and T5, T10, and T15 at 1 mA.
Wiethoff et al., 2014 [27]	53	10 minutes; 5x7cm ; 2 mA	target: left M1 (FDI hotspot); ref: right supraorbital	atDCS, ctDCS	MEPs (pre-tDCS, T0, T5, T10, T15,	1 mV baseline; 30 MEPs per session; pulses every 4.5 -	(i) grand average: CSE >1 increase, <1 reduction;	47.2 % atDCS (ctDCS 52.8% but facilitation)	At the group level, atDCS increased MEPs while ctDCS did not affect CSE. Cluster analysis revealed two clusters for each stimulation condition. Half of the participants showed a facilitatory effect in both atDCS, and ctDCS conditions, and the other half showed no response.

					T20, T25, T30)	5.5 s; right FDI	(ii) two steps cluster analysis		

The table includes studies investigating the prevalence of responders and non-responders in their samples. Scrolling the table from left to right, for each study, we summarized the number of participants (N), stimulation protocols including duration, electrodes size (or areas), current intensity, electrodes position, the stimulation type (b=between participants condition, w=within participants condition), the recorded measure/dependent variable. In most studies, MEPs were recorded; therefore, the TMS protocol was also included. We then summarized the responders' determination criterion and percentage and concluded with the papers' results.

ACC= accuracy; ADM = abductor digiti minimi; atDCS = anodal tDCS; CSE = corticospinal excitability; ctDCS = cathodal tDCS; DSB = digit span backward; FDI = first dorsal interosseous; ICF = intracortical facilitation; IO-curves = input-output curves; M1 = primary motor cortex; MEPs = motor evoked potentials; NR = not reported; rMT = resting motor threshold; RTs = reaction times; SD = standard deviation; SEM = standard error of the mean ; SICI = short-latency intracortical inhibition; T(number) = indicates the minutes after stimulation at which the dependent variable was recorded.

[a] 0.5 mA intensity was repeated in two different sessions to measure intra-individual variability
[b] 34 participants received both atDCS and ctDCS, 2 additional participants received only atDCS
[c] in the sham condition, participants received 0.1 mA for 20 minutes
[d] participants were older (mean age =66.85), categorized for the BDNF Val66Met Polymorphism in homozygotes for the Val allele (n=13) or Met carriers (n=37).

Table S2 summarizes the studies in which stable individual features, namely anatomical, neurochemical, and demographical characteristics and genotype, were used to investigate tDCS effects. Electrode configuration is reported using EEG 10-20 references; target electrode (mainly anode) is reported before the reference electrode (mainly the cathode) (i.e., target electrode + reference electrode).

A) Stable Factors: skull thickness, cortex morphology, and gyrification									
**Study**	**Factor**	**Study Design**	**N**	**Stimulation protocol**	**Electrodes' montage (target+ref. electrode) and dimension**	**Target area**	**Task**	**Outcome Measures**	**Results**
Datta et al., 2009 [53]	Skull thickness	Simulation study: a realistic head model derived from structural MRI	-	Single session of a-tDCS and a-HD-tDCS at 1 mA and 2 mA	Electrode configurations: (1) left M1 + contralateral SO; electrode size: 35 cm^2; (2) 4×1 HD-tDCS electrode in a circular fashion around the anode over M1.	motor cortex	-	Electric field distribution	Skull and CSF thickness are the most responsible for changes in EF magnitude reaching the cortex.
Datta et al., 2010 [58]	Skull thickness	Simulation study: a realistic head model derived from structural MRI compared to a realistic brain-injured model (with different defects configuration – skull plates).	-	Single session of a-tDCS at 1 mA	Electrode configurations: (1) C3 + contralateral SO; (2) O1 + contralateral SO. Electrode size: 35 cm^2	motor cortex	-	Electric field distribution	The distance between the electrode and the skull defect and the conductivity of the skull plates influence the EF.
Datta et al., 2011 [71]	Cortex morphology	Simulation study: a realistic head	-	Single session of a-tDCS at 1 mA	Four configurations: (1,2,3) damaged	frontal cortex	-	Electric field distribution	The reference electrode's position

		model derived from structural MRI of a 60 y.o. aphasic male with a left hemisphere ischemic stroke (lesion size 5 87.42 mL)			left frontal cortex + right shoulder / right mastoid / right SO;<br>(4) ‘‘mirror’’ configuration (undamaged right cortex + right shoulder).<br>Electrode size: 25 cm^2.				affected brain current flow and EF in the perilesional and wider cortical lesions.
Datta, 2012 [50]	Cortex morphology	Simulation study: realistic head models derived from structural MRI of one 36 y.o. male; one 41 y.o. male, and one 34 y.o. female	-	Single session of a-tDCS and a-HD-tDCS at 1 mA	Electrode configurations:<br>(1) left M1 + contralateral SO; electrode size: 25 cm^2 ;<br>(2) 4 × 1 HD-tDCS electrode in a circular fashion around the anode over M1	motor cortex	-	Electric field distribution	Cortical gyri/sulci details influence the current flow profile across all simulations.
Filmer et al., 2019 [70]	Cortex morphology	Real tDCS study	47 subjects (26 females; mean age=22).	Three 9 min offline sessions of a-tDCS, c-tDCS and sham at 0.7 mA	Electrode configuration: 1 cm posterior to F3 + 1 cm posterior to F4. Electrode size: target 5x5 cm^2 and ref 5x7 cm^2	DLPFC	sensory-motor decision-making task	Reaction times	Cortical thickness of the lDLPFC accounted for almost 35% of the inter-individual difference in behavioral performance for atDCS. No differences were traceable for c-tDCS.

Hanley & Tales, 2019 [57]	Skull to cortex distance, ageing	Real tDCS study	24 older subjects (16 females; range: 54–75 y.o; mean age= 66.46 ± 5.34)	Three 20 min offline sessions of a-tDCS and sham at 1.5 mA	Electrode configuration: F3 + F4. Electrode size: 25 cm^2.	DLPFC	task switching paradigm (Swansea Test of Attentional Control, STAC)	Reaction times	AtDCS improves attentional control in older adults.
Indahlastari et al., 2020 [60]	Skull to cortex distance	Simulation study: realistic head models from 587 healthy older adults (range: 51-95 y.o., mean=73.9)	-	Single session of a-tDCS at 2 mA	Two configurations: (1) F3 + F4; (2) M1 + SO. Electrode size: 35 cm^2.	motor cortex; DLPFC	-	Electric field distribution	Brain atrophy negatively correlates to EF. The brain-to-CSF ratio partially mediates the negative correlation between age and CD.
Laakso et al., 2015 [54]	Skull to cortex distance	Simulation study: realistic head models derived from structural MRI of 24 males	-	Single session of a-tDCS at 1 mA	Electrode montage: C3 + Fp2. Electrode size: 35 cm^2	motor cortex	-	Electric field distribution	Age has a slightly negative effect on EF. A thicker layer of CSF decreases the electric field strength, explaining 50% of the inter-individual variability.
Mahdavi & Towhidkhah, 2018 [56]	Skull to cortex distance	Simulation study: three high-resolution human head models representing	-	Single session of a-tDCS at 1 mA	Two configurations: (1) T3 + SO; (2) F3 + SO. Electrode size: 35 cm^2	motor cortex	-	Electric field distribution	CD decreased with decreasing GM volume in MCI and healthy aging compared to

		young, elder, and mild cognitive impaired (MCI) subjects							the young model. CD flows in the depth of cortical regions by CSF in case of morphology alterations of cerebral sulci.
Metwally et al., 2015 [64]	Cortex morphology	Simulation study: a realistic head model derived from structural MRI of a 38 y.o. Asian male	-	Single session of a-tDCS and a-HD-tDCS at 0.5 mA, 1 mA and 2 mA	Three configurations: (1) C3 + right SO; (2) C3 + C4; (3) 4x1 HD-tDCS ring electrodes. Electrode size: 35 cm^2.	Motor cortex	-	Electric field distribution	Brain tissue anisotropy affects both distribution and magnitude of EF. Skull anisotropy influences EF radial distribution. WM anisotropy strongly alters the EF directionality, especially within the sulci.
Miranda et al., 2006 [52]	Skull thickness	Simulation study: a realistic head model	-	Single session of a-tDCS at 2 mA	Four configurations: (1) left M1 + contralateral SO; (2) left DLPFC + contralateral SO; (3) symmetrically supraorbital (147 mm anterior to Cz) + two small	motor cortex	-	Electric field distribution	Skull thickness is the primary factor responsible for EF attenuation.

					cathodes placed over the mastoids; (4) premotor cortex + two small cathodes placed over the mastoids. Electrode size: 25 cm^2 and 6.5x15 cm^2				
Miranda et al., 2013 [49]	Cortex morpholo gy	Simulation study: a realistic head model derived from structural MRI	-	Single session of a-tDCS and a-HD-tDCS at 1 mA	Electrode configuration: right M1 + contralateral SO. Three different anode sizes: 5×7 cm^2, 3×3 cm^2, 1 cm circular.	Motor cortex	-	Electric field distribution	The cortical response is maximized when the M1 hotspot is in a sulcus instead of a gyrus. Smaller electrodes improved the focality of the tangential but not the normal EF component.
Opitz et al., 2015 [43]	Skull thickness	Simulation study: realistic head models derived from structural MRI of one 27 y.o. male and one 26 y.o. female	-	Single session of a-tDCS at 1 mA	28 different electrode montages were obtained by rotating and moving the anode in anterior-posterior and medial-lateral steps of 5 mm over the left M1 + contralateral SO. Electrode size: 35 cm^2	motor cortex	-	Electric field distribution	Skull and CSF thickness and sulcal depth explain 50% of the variability in EF magnitude reaching the cortex.

Rawji et al., 2018 [72]	Cortex morphology	Real tDCS study	22 healthy adults (17 males; aged 21-44; mean age= 28.95 ± 6.14)	Two 10 min offline sessions of a-tDCS and sham at 1 mA	Electrode configurations 7 cm anterior or posterior to the left M1 – FDI hotspot: (1) a-anterior + c-posterior; (2) c-anterior + a-posterior; (3) a-medial + c-lateral.	motor cortex	-	MEPs	Orthogonal but not parallel-orientated tDCS modulates MEPs.
Russell et al., 2013 [66]	Cortex morphology	Simulation study: realistic head models derived from structural MRI of 18 subjects (10 males) aged from 21 to 68 y.o.	-	Single session of a-tDCS at 2 mA	Four configurations: (1) F8 + P2; (2) C3 + C4; (3) F7 + F8; (4) FPz + O1. The virtual electrodes were a single voxel in size.	motor cortex; primary visual cortex; DLPFC	-	Electric field distribution	Significant variation occurs between subjects with the same applied electrode configuration with ten-fold differences near the electrode and two-fold individual differences in sites distant from the electrodes.
Seo et al., 2017 [47]	Cortex morphology	Simulation study: a realistic head model derived from structural MRI	-	Single session of a-HD-tDCS at 1 mA	4×1 HD-tDCS: target hand knob in the precentral gyrus55 + 4 return electrodes in a circular fashion.	Motor cortex	-	Electric field distribution	Morphology and gyrification affect tDCS-induced membrane polarization.

Shahid et al., 2014 [67]	Cortex morphology	Simulation study: a realistic head model derived from the publically available dataset (the BrainWeb)	-	Single session of a-tDCS at 1 mA	Four configurations: (1) C3 + Fp2; (2) F3 + Fp2; (3) P3 + Fp2; (4) C3 + C4. Electrode size: 25 cm^2.	DLPFC, motor cortex, parietal cortex (F3, C3, P3)	-	Electric field distribution	Anisotropy in the GM and subcortical regions affect the strength and spatial distribution of the induced EF. The inclusion of GM and subcortical anisotropy increased the average percentage difference in the Ef strength of motor cortex to 34%, with respect to 5% of WM anisotropy only.
Suh et al., 2012 [51]	Skull thickness	Simulation study: a realistic head model derived from structural MRI	-	Single session of a-tDCS at 1 mA	Electrode configuration: C3 + C4. Electrode size: rounded electrode 8 mm diameter.	motor cortex	-	Electric field distribution	Anisotropic skull conductivity affects the CD and EF distribution by 12-14%. WM anisotropy does not significantly influence the CD and EF on the targeted

									cortical surface.
Sun et al., 2021 [45]	Skull thickness	Simulation study: a realistic head model derived from structural MRI of a 25y old Asian male	-	Single session of a-tDCS at 1 mA and a-HD-tDCS at 1 mA (~0.25 mA each)	Three configurations: (1) Cz / FCz / C1 / FC1 + Fp2; (2) Cz / CPz / C2 / CP2 + Fp1. Electrode size: 8 cm^2. (3) 4x1 HD-tDCS.	motor cortex	-	Electric field distribution	Distinguishing the skull into compact bone and spongy bone is important for an accurate tDCS simulation.
Truong et al., 2013 [203]	Skull thickness – fat	Simulation study: realistic head models derived from structural MRI of a 35 y.o. female, a 47 y.o. female, a 22 y.o. female, a 36 y.o. male, and a 25 y.o. female with different BMI from obese to normal	-	Single session of a-tDCS and a-HD-tDCS at 1 mA	Three configurations: (1) C3 + contralateral SO; (2) F8 + contralateral SO. Electrode size: 35 cm^2 (3) 4×1 HD-tDCS electrode in a circular fashion around the anode over C3.	motor cortex		Electric field distribution	Head fat has a minor influence on CD across the brain.
Unal et al., 2020 [63]	Skull to cortex distance	Simulation study: realistic head models of the three variants of the primary progressive aphasia (nfvPPA,	-	Single session of a-tDCS and a-HD-tDCS at 2 mA	Two configurations: (1) F7 + right cheek; electrode size: 25 cm^2; (2) 4 × 1 HD-tDCS montage.	DLPFC	-	Electric field distribution	Local brain atrophy alone does not predict local EF.

		svPPA, lvPPA)							
Wagner et al., 2007 [59]	Cortex morphology	Simulation study: a derived healthy head model vs. three different stroke models	-	Single session of a-tDCS and c-tDCS at 1 mA	Six configurations: (1) right M1 + left SO; electrode size: 7x7; 7x5; 5x5; 1x1 cm^2; (2) right M1 + left M1; (3) V1 + vertex; (4) V1 + left SO; (5) DLPFC + SO; (6) right DLPFC + left DLPFC. Electrode size: 5x7 cm^2.	motor cortex; primary visual cortex; dorsolateral prefrontal cortex	-	Electric field distribution	Pathological models have elevated CD maxima and an altered location relative to the non-pathological models.
Wagner et al., 2014 [74]	Cortex morphology, Skull thickness	Simulation study: a realistic head model derived from structural MRI of a healthy 26 y.o. male	-	Single session of a-tDCS at 1 mA	Two configurations: (1) right auditory cortex + homologous left; (2) left M1 + contralateral SO. Electrode size: 35 cm^2.	motor cortex, auditory cortex	-	Electric field distribution	Skin, spongy skull, and CSF affect EF distribution. Current vectors tend to be oriented towards the closest higher conducting region. Anisotropic WM conductivity causes current flow in directions parallel to the WM fiber tracts. The highest cortical current magnitudes

									are found not only close to the stimulation sites. The median brain CD decreases with increasing distance from the electrodes.
B) Stable factors: neurochemical features									
**Study**	**Factor**	**Study Design**	**N**	**Stimulation protocol**	**Electrodes' montage (target+ref. electrode) and dimension**	**Target area**	**Task**	**Outcome Measures**	**Results**
Fresnoza et al., 2014 [82]	dopamine	Real tDCS study	12 healthy subjects (7 males; mean age= 27.92 ± 1.60 y.o.)	Two sessions of 13 min a-tDCS and 9 min c-tDCS at 1 mA	Electrode configuration: left M1 (right ADM hotspot) + contralateral SO. Electrode size: 35 cm^2.	motor cortex	No task. Intervention: placebo or 2.5, 10, 20 mg bromocriptine somministration.	MEPs	Modulation of D2-like receptor activity exerts a non-linear dose-dependent effect on neuroplasticity in M1.
Monte-Silva et al., 2010 [83]	dopamine	Real tDCS study	12 healthy adults (5 men; mean age= 30.83 ± 5.10)	Two sessions of 13 min a-tDCS and 9 min c-tDCS at 1 mA	Electrode configuration: left M1 (right ADM hotspot) + contralateral SO. Electrode size: 35 cm^2.	motor cortex	No task. Intervention: placebo or 25, 100, 200 mg L-dopa somministration.	MEPs	Dopamine has non-linear dosage-dependent effects on both atDCS and ctDCS induced plasticity.
Stagg et al., 2009 [78]	GABA and glutamate	Real tDCS study	25 healthy volunteers (8 males; range	Three 10 min sessions of a-tDCS, c-tDCS	Electrode configuration: C3 + contralateral SO.	motor cortex	-	neurotransmitters	A-tDCS locally reduces

			20–49 y.o.) in total	and sham at 1 mA	Electrodes size: 35 cm^2.			concentration	GABA and c-tDCS reduces glutamatergic neuronal activity with a highly correlated reduction in GABA.
Stagg et al., 2014 [80]	GABA and glutamate	Real tDCS study	50 healthy volunteers (17 males; younger range between 20–39 y and older between 45–72 y)	Single 10 min session of a-tDCS at 1 mA	Electrode configuration: C3 + contralateral SO. Electrode size: 35 cm^2.	motor cortex	-	neurotransmitters concentration	Network-level functional connectivity within the motor system is related to the degree of inhibition in M1. A-tDCS decreases GABA levels within M1 and increases resting motor network connectivity.
C) Stable Factors: genetic profile									
**Study**	**Factor**	**Study Design**	**N**	**Stimulation protocol**	**Electrodes' montage (target+ref. electrode) and dimension**	**Target area**	**Task**	**Outcome Measures**	**Results**
Antal et al., 2014 [87]	BDNF Val/Val vs. Met Carrier	Real tDCS study	64 healthy subjects (34 females; age range: 19-40 years)	Single session for each participant of 7, 10 or 13 min of a-tDCS; 9 or 10 min of c-tDCS at 1 mA and	Electrode configuration: left M1 + contralateral SO. Electrode size: 35 cm^2. tACS electrode size: 4x4 and 14x6 cm.	motor cortex	-	MEPs	No differences between the two allele groups that show: - increased MEPs

				10 min of tACS at 140 Hz.					compared to baseline after a-tDCS and tACS;<br>- reduced MEPs compared to baseline after c-tDCS.
Moliadze et al., 2014 [231]	BDNF Val/Val homozygotes	Real tDCS study	12 healthy subjects (mean age: 25.7 ± 4.1 years; range: 23–38 years)	Four experiment condition with 10 min sessions of a-tDCS, sham and tRNS at 1 mA	Electrode configuration: left M1 + contralateral SO. Electrode size: anode 16 cm^2 and cathode 84 cm^2.	motor cortex	-	MEPs	Val/Val carriers show a significant increase in MEP after a-tDCS compared to sham stimulation.
Nieratschker et al., 2015 [88]	COMT Val/Val vs. Met Carrier	Real tDCS study	41 healthy subjects (32 females; mean age=24.0 ± 4.2)	Single session of 20 min of c-tDCS at 1 mA and sham	Electrode configuration: F3 + right SO. Electrode size: 35 cm^2.	DLPFC	go/no-go task	Accuracy and RTs	Genetic factors modulate the effects of tDCS on cognitive performance. C-tDCS has a detrimental effect on response inhibition only in individuals homozygous for the Val-allele of the COMT. No effects were traceable in the Met-allele carriers.

Plewnia et al., 2013 [89]	COMT Val/Val vs. Met Carrier	Real tDCS study	46 healthy subjects (21 females; mean age=25.87 ±, 7.29)	Single session online of 20 min of a-tDCS at 1 mA and sham	Electrode configuration: F3 + right SO. Electrode size: 35 cm^2.	DLPFC	Parametric Go/no-Go test	Accuracy and RTs	Genetic factors modulate the effects of tDCS on executive functions. The COMT Met/Met allele carrier predicts a detrimental effect of a-tDCS on cognitive flexibility.
Puri et al., 2015 [33]	BDNF Val/Val vs. Met Carrier	Real tDCS study	54 healthy older adults (mean age = 66.85 years)	Two sessions of 10 and 20 min of a-tDCS at 1.5 mA	Electrode configuration: right FDI area + contralateral SO. Electrode size: anode 25 cm^2 and cathode 51 cm^2.	motor cortex	-	MEPs	Met carriers have a significant increase in MEPs after 20 min of a-tDCS compared to Val/Val. No significant effect after 10 min of a-tDCS.
Teo et al., 2014 [85]	BDNF Val/Val vs. Met Carrier	Real tDCS study	58 healthy subjects	Single session of 9 min a-tDCS at 1 mA	Electrode configuration: left M1 (right FDI hotspot) + contralateral SO. Electrode size: 35 cm^2.	motor cortex	-	MEPs	Met carriers show a significant increase in MEPs between 30 and 90 min from a-tDCS.
D) Stable Factors: age and gender effects									

Study	Factor	Study Design	N	Stimulation protocol	Electrodes' montage (target+ref. electrode) and dimension	Target area	Task	Outcome Measures	Results
Bhattacharje et al., 2022 [92]	Age and gender on skull thickness	Simulation study: realistic head models derived from structural MRI of 240 healthy subjects (120 males; range: 18-87 y.o.)	-	Single session of a-tDCS at 2 mA	Two configurations: (1) CP5 + Cz; (2) F3 + Fp2. Electrode size: 25 cm^2.	inferior parietal lobule, middle frontal gyrus	-	Electric field distribution	Gender differences interact with age and stimulation targets in modulating the simulated CD of tDCS.
Farcito et al., 2019 [90]	Age and gender on skull thickness	Simulation study: realistic head models derived from structural MRI of 20 healthy subjects stratified by age, sex, and ancestry	-	Single session of a-tDCS at 1 mA	Electrode configuration: C3 + C4. Electrode size: 35 cm^2.	Motor cortex	-	Electric field distribution	Higher CD in the skull and cortex around areas where the spongy bone layer is thickest. Locally higher CD in the area near veins results from current shunting through the CSF layer under the vein, thus flowing closer to the cortex.
Fujiyama et al., 2014 [96]	Age and genotype	Real tDCS study	40 healthy subjects: older adults (13 females; mean age=68.3±7.9	Two 30 min sessions of a-tDCS and sham at 1 mA	Electrode configuration: left FCR area + contralateral SO. Electrode size:	motor cortex	-	MEPs	In the younger group, CSE significantly increased immediately

			y.o.) vs. 20 young adults (10 females; mean age= 22.7±3.3 y.o.)		anode 25 cm^2; cathode 51 cm^2.				after stimulation. In older, CSE increased only 20 min after stimulation. BDNF genotype did not result in significant differences for either age group.
Hunold et al., 2021 [93]	Age on skull thickness	Simulation study: realistic head models derived from structural MRI of 20 healthy subjects stratified by age (4 children 10.95±1.32; 8 adolescents 15.10±1.16; 8 young adults 21.62±2.45)	-	Single session of a-tDCS at 1 mA	Electrode configuration: left M1 + right SO. Electrode size: 35 cm^2.	Motor cortex, DLPFC	-	Electric field distribution	Skull thickness increased with age. Cortical CD magnitude is higher in children than adults for a given tDCS current strength.
Kessler et al., 2013 [94]	Children	Simulation study: realistic head models derived from structural MRI of 3 healthy adults (2	-	Single session of a-tDCS and a-HD-tDCS at 1 mA and 2 mA	Four tDCS configurations: (1) M1 + contralateral SO; (2) C3 + C4; (3) TP7 + TP8; (4) F3 + F4. Two 4x1 HD-tDCS electrode	motor cortex, DLPFC, parietal cortex	-	Electric field distribution	Children are exposed to higher peak EF for a given current intensity than adults. Similarities exist between

		females of 25 and 33 y.o., 1 male 36 y.o.) and 2 children (8 and 12 y.o.)			configurations: 5 cm separation and 2.5 cm separation.				adults with smaller head sizes and children.
Kuo et al., 2006 [107]	Gender	Real tDCS study	66 females (26.2±2.2) vs. N=52 males (27.4±3.9 y.o.)	Single session of 13 min a-tDCS and 9 min c-tDCS at 1 mA	Electrode configuration: right ADM area + contralateral SO. Electrode size: 35 cm^2.	motor cortex	-	MEPs	A-tDCS significantly increased MEPs compared to baseline with no significant difference between genders. C-tDCS reduced MEPs compared to baseline, with reductions significantly greater in females than males.
Moliadze et al., 2015 [95]	Children	Real tDCS study	19 healthy children (11 girls; mean age= 13.9±0.4 years; ranege: 11–16 years)	Two experiment condition with 10 min single sessions of a-tDCS, c-tDCS and sham at 0.5 mA and 1 mA	Electrode configuration: left M1 + right SO. Electrode size: 35 cm^2.	motor cortex	-	MEPs	In children:<br>- 0.5 mA c-tDCS decreases CSE<br>- 0.5 mA a-tDCS had no effect<br>- both 1 mA a- and c-tDCS resulted in a significant increase of MEPs.

Russell et al., 2014 [91]	Gender on skull thickness	Simulation study: realistic head models derived from structural MRI of 24 healthy adults (12 males with a mean age of 53 ± 11.5 y.o. and 12 females with a mean age of 50.5 ± 14.3)	-	Single session of a-tDCS at 0.5 mA, 1 mA and 2 mA	Two configurations: (1) C3 + C4; (2) F3 + F4. Electrodes size: 20 mm round and 22.5 cm^2.	motor cortex, DLPFC	-	Electric field distribution	Males have spongier parietal bones, while females have denser parietal bones. Females received significantly less current at the targeted parietal cortex than males at the same current intensity. Electrodes in the frontal regions conducted less than those in the parietal region.

The table includes the studies in which stable individual features, namely anatomical, neurochemical, and demographical characteristics and genotype, were used to investigate tDCS effects. Scrolling the table from left to right, for each study, we summarized the investigated feature, the study design (real or simulated), the number of participants (N), stimulation protocols including duration and current intensity, electrodes position, the targeted brain area, the task performed by participants, the outcome measure (included MEPs) and the study conclusion. Electrode configuration is reported using EEG 10-20 references; target electrode (mainly anode) is reported before the reference electrode (mainly the cathode) (i.e., target electrode + reference electrode).

ADM= abductor digit minimi; a-tDCS= anodal tDCS; CD= current density; CSE= cortico-spinal excitability; CSF= cerebrospinal fluid; c-tDCS= cathodal tDCS; DLPFC= dorsolateral prefrontal cortex; EF= electric field; FCR= flexor carpi radialis; FDI= first dorsal interosseous; GM= gray matter; HD-tDCS= high definition tDCS; ICF= short latency intracortical facilitation; MEPs= motor evoked potentials; M1= primary motor cortex; MRS= magnetic resonance spectroscopy; PFC= prefrontal cortex; RTs= reaction times; SICI= short latency intracortical inhibition; SO= supraorbital area; WM= white matter

Table S3 summarizes the studies in which individual variable features, namely individual sensitivity and substance assumption, were used to investigate tDCS effects. Electrodes' configuration is reported using EEG 10-20 references; target electrode (mainly anode) is reported before the reference electrode (mainly the cathode) (i.e., target electrode + reference electrode).

Variable physiological factors									
**Study**	**Factor**	**Study Design**	**N**	**Stimulation protocol**	**Electrodes' montage (target+ref. electrode) and dimension**	**Target area**	**Task**	**Outcome Measures**	**Results**
Batsikadze et al., 2015 [120]	Nicotine	Real tDCS study	12 healthy non-smokers subjects (8 females; 24.4 ± 4.7 y.o.) took part to tDCS experiment	Single session of 13 min a-tDCS and 9 min c-tDCS at 1 mA	Electrode configuration: left M1 (right ADM hotspot) + right SO. Electrode size: 35 cm^2.	motor cortex	No task. Intervention: low (0.1 mg), medium (0.3 mg), or high (1.0 mg) dosages of varenicline or 0.5 mg placebo 3h before stimulation.	MEPs	Activation of nicotinic α4β2 has specific and dosage-dependent effects on neuroplasticity in healthy, non-smoking individuals. Low-dose varenicline had no impact on tDCS-induced neuroplasticity; medium-dose abolished tDCS-induced facilitatory after-effects; high-dose preserved c-tDCS-induced excitability diminution and focal excitatory PAS-induced facilitatory plasticity.
Grundey et al., 2012 [121]	Nicotine	Real tDCS study	24 young, non-smoking	Single session of 13 min a-tDCS,	Electrode configuration: left M1 (right	motor cortex	No task. Intervention: placebo or	MEPs	Nicotine spray affects neuroplasticity in

			healthy subjects (13 females)	9 min c-tDCS and PAS at 1 mA	ADM hotspot) + right SO. Electrode size: 35 cm^2.		nicotine spray.		non-smoking subjects. tDCS-derived excitability reduction was delayed and weakened by nicotine. This effect could differ from those of prolonged nicotine application.
Grundey et al., 2018 [122]	Nicotine	Real tDCS study	12 young, healthy subjects	Single session of 13 min a-tDCS at 1 mA	Electrode configuration: left M1 (right ADM hotspot) + right SO. Electrode size: 25 cm^2.	motor cortex	No task. Intervention: placebo or nicotine and flunarizine.	MEPs	Abolished tDCS-induced neuroplasticity because of nicotine administration is reversed by calcium channel blockade with flunarizine in a dose-dependent manner.
Labruna et al., 2019 [106]	Individual sensitivity	Real tDCS study	42 young adults (18 females; mean age: 22.2 ± 3.8 y.o.)	Single session of 20 min online a-tDCS and sham at 2 mA	Electrode configuration: left M1 (FDI hotspot) + Fp2. Electrode size: 25 cm^2.	motor cortex	Visuomotor adaptation (VMA) task	MEPs, Angular error, Movement time	TMS sensitivity can predict tDCS efficacy in a behavioral task.
Lattari et al., 2019 [118]	Caffeine	Real tDCS study	15 healthy young males (mean age: 25.3±3.2 y.o.)	Four experimental conditions with20 min a-tDCS and sham at 2 mA	Electrode configuration: left DLPFC.	DLPFC	No task. Intervention: caffeine or placebo 1h before stimulation.	Muscular strength, Perceived exertion rating	Caffeine associated with a-tDCS reduced perceived exertion rating compared with placebo. Both caffeine and a-tDCS increased muscle strength.
Nitsche et al., 2009 [134]	Serotonine	Real tDCS study	12 healthy subject (4 females;	Single session of 13 min a-tDCS,	Electrode configuration: left M1 (right	motor cortex	No task. Intervention: 20 mg	MEPs	Citalopram enhanced and prolonged the

			mean age 25.1 ± 1.16 y.o.).	9 min c-tDCS at 1 mA	ADM hotspot) + right SO. Electrode size: 35 cm^2.		citalopram or placebo 2 h before stimulation.		facilitation induced by anodal tDCS, whereas it turned cathodal tDCS-induced inhibition into facilitation.
Nitsche, Grundey, et al., 2004 [124]	Amphetamine	Real tDCS study	12 young, healthy subject	Single session of 7, 13 min a-tDCS, 9 min c-tDCS at 1 mA	Electrode configuration: left M1 (right ADM hotspot) + right SO. Electrode size: 35 cm^2.	motor cortex	No task. Intervention: 20 mg amphetaminil (AMP), 80 mg PROP a combination of 20 mg AMP and 150 mg DMO or placebo.	MEPs	Amphetamine significantly enhanced and prolonged a-tDCS-induced long-lasting excitability.
Nitsche, Liebetanz, et al., 2004 [132]	GABAergic modulation	Real tDCS study	12 young, healthy subjects	Single session of 5, 11 min a-tDCS, 9 min c-tDCS at 1 mA	Electrode configuration: left M1 (right ADM hotspot) + right SO. Electrode size: 35 cm^2.	motor cortex	No task. Intervention: 20 mg lorazepam or placebo 2h before stimulation.	MEPs	The GABAA receptor agonist lorazepam administration resulted in a delayed but enhanced and prolonged anodal tDCS induced excitability elevation.
Thirugnanasambandam et al., 2011 [119]	Nicotine	Real tDCS study	24 healthy non-smokers took part to tDCS experiment	Single session of 13 min a-tDCS and 9 min c-tDCS at 1 mA	Electrode configuration: left M1 (right ADM hotspot) + right SO. Electrode size: 35 cm^2.	motor cortex	No task. Intervention: nicotine patches or placebo.	MEPs	Nicotine abolished or reduced both PAS- and tDCS-induced inhibitory neuroplasticity. Non-focal facilitatory plasticity was also abolished, whereas focal plasticity was

									slightly prolonged by nicotine.

The table includes the studies in which stable individual features, namely individual sensitivity and substance assumption, were used to investigate tDCS effects. Scrolling the table from left to right, for each study, we summarized the investigated feature, the study design (real or simulated), the number of participants (N), stimulation protocols including duration and current intensity, electrodes position, the targeted brain area, the task performed by participants, the outcome measure (included MEPs) and the study conclusion. Electrode configuration is reported using EEG 10-20 references; target electrode (mainly anode) is reported before the reference electrode (mainly the cathode) (i.e., target electrode + reference electrode).

ADM= abductor digit minimi; a-tDCS= anodal tDCS; CD= current density; CSE= cortico-spinal excitability; CSF= cerebrospinal fluid; c-tDCS= cathodal tDCS; DLPFC= dorsolateral prefrontal cortex; EF= electric field; FCR= flexor carpi radialis; FDI= first dorsal interosseous; GM= gray matter; HD-tDCS= high definition tDCS; ICF= short latency intracortical facilitation; MEPs= motor evocked potentials; MRS= magnetic resonance spectroscopy; M1= primary motor cortex; PFC= prefrontal cortex; RTs= reaction times; SICI= short latency intracortical inhibition; SO= supraorbital area; WM= white matter.

Table S4 summarizes the studies in which baseline participants' performance was used to investigate tDCS effects.

Study	N	Protocol	Electrodes' placement	Stimulation type	tDCS - task time-locking	Task	Outcome measures	Baseline determination	Results
Benwell et al., 2015 [41]	38	20 minutes; 4x4 cm electrodes; intensity: half sample 1 mA, the other 2 mA	bihemispheric PPC (p5, p6)	LA-RC, LC-RA, sham (w)	online (task performance pre - during - post tDCS)	perceptual line bisection	point of subjective equality	High vs. low discrimination sensitivity groups based on the average performance in the pre-stimulation session.	Rightward shift in subjective midpoint after LA-RC. The effect was weak over the entire sample. Subsets of participants responded differently according to their baseline and current intensity. 1 mA tDCS induced a rightward shift in the high sensitivity group, 2 mA in the low.
Gözenman & Berryhill, 2016 [160] - Study 1	24	20 minutes; atDCS:5x7 cm; aHD-tDCS ~0.5"; 1.5 mA	target: rPPC (p4); atDCS ref: left cheek	atDCS, HD-tDCS, sham (w)	online	retro-cue task	accuracy, RTs	High vs. low performers groups based on their Ospan in the pre-stimulation session.	Low performers improved after HD-tDCS and worsened after tDCS. No difference for high performers.
Gözenman & Berryhill, 2016 [160] - Study 2	24	20 minutes; atDCS:5x7 cm; aHD-tDCS ~0.5"; 1.5 mA	target: lPPC (p3); atDCS ref: right cheek	atDCS, HD-tDCS, sham (w)	online	retro-cue task	accuracy, RTs	High vs. low performers groups based on their Ospan in the pre-stimulation session.	No significant interaction between tDCS condition and performers level.
Heinen et al., 2016 [158] – Study 3	16	20 minutes; 4.5 x 6.5; 1.5 mA	Target: rPPC (P4); ref: left arm	atDCS, ctDCS, sham (w)	online	visual working memory task	Accuracy (WM precision and several error types	Correlation between pre-stimulation and stimulation session.	No effect at the group level. ctDCS benefitted low performers but did

							separately analyzed)		not affect high performers.
Hsu et al., 2014 [161]	20	15 minutes; 4x4 cm electrodes; 1.5 mA	target: rPPC (p4); ref: left cheek	atDCS, sham (w)	offline	change detection task	sensitivity indexes (d', K), EEG recordings	High vs. low performers groups based on the change of the detection task performance at sham.	Low performers benefitted from atDCS at behavioral and electrophysiological levels.
Hsu et al., 2016 [152]	18	15 minutes; 4x4 cm electrodes; 1.5 mA	target: rPPC (p4); ref: left cheek	anodal, cathodal, sham (w)	offline	Corsi tapping task	sensitivity indexes (d', K)	High vs. low performers based on pre-stimulation performance at digit span forward or Corsi tapping task.	Low performers worsened after atDCS but only when groups were divided based on their performance at the Corsi tapping task. No differences were traceable for high vs. low performers based on the digit span.
Jones & Berryhill, 2012 [159] - Study 1	20	10 minutes; 5x7 cm electrodes; 1.5 ma	target: rPPC (P4); ref: left cheek	atDCS, ctDCS, sham (w)	offline	change detection task, sequential presentation task	accuracy	High vs. low performers groups based on the pre-stimulation combined score of digit span forward and backward.	No tDCS effects at the group level. AtDCS and ctDCS improved performance in the high-capacity group and worsened it in the low. These differences emerged only in the change detection task (the most challenging task).
Jones & Berryhill, 2012 [159] - Study 2	28	10 minutes; 5x7 cm electrodes; 1.5 ma	target: rPPC (P4); ref: left cheek	atDCS, ctDCS, sham (w)	offline	change detection task (different set sizes)	accuracy	Digit span forward and backward combined score. Difficulty of	Both tDCS conditions improved performance in the high WM capacity group, and this benefit was more

								the change detection task was parametrically increased by varying the set size.	significant at higher task difficulty levels. Conversely, the low WM capacity group was unaffected by stimulation.
Learmonth et al., 2015 [151]	40[a]	15 minutes; anode 5x5, cathode 5x7; 1 mA	Targer: lPPC (P3) or rPPC (P4); ref: contralateral supraorbital area	anodal, sham (w)	online	titrated lateralized visual detection task;	Task sensitivity (d')	High vs. low performers groups based on the task performance in the pre-stimulation block (block 1)	Poor performers were impaired by lPPC stimulation compared to sham; good performers maintained good sensitivity in rPPC stimulation
London & Slagter, 2020 [167]	34	20 minutes; 5x7 cm electrodes; 1 mA	target: lDLPFC (F3); ref: contralateral supraorbital region	atDCS, ctDCS (w)	online (task performance pre - during - post tDCS)	Rapid serial visual presentation of letters	attentional blink magnitude, T2 priming	Correlation of task pre-stimulation performance with during and post-stimulation conditions.	No effects at the group level nor considering individuals' baseline performance.
Mizuguchi et al., 2018 [157]	23	20 minutes; 5x5 cm electrodes; 2 mA	target: right cerebellum; right buccinator muscle	atDCS, ctDCS, sham (w)	online (block 1 before tDCS; blocks 2 - 5 during tDCS; block 6 after stimulation)	Dart throwing (motor task)	Performance index: distance between the center of the bull's eye and the dart's stick point.	Correlation between task performance in the first block of each session and the improvement during each stimulation condition.	No tDCS effects at the group level. Low performers showed a greater improvement in the ctDCS condition than the sham. No differences were found in the good performers' group.
Reinhart et al., 2016 [168] - Study 4	20	20 minutes; target: 19.25 cm2, ref: 52 cm2; 2 mA	target: occipital cortex - hemisphere counterbalanced across participants- (P1-P2) -; ref:	atDCS, sham	offline	Snellen acuity task	the logarithm of the minimal angle of resolution	Correlation between the task performance at baseline and after stimulation.	AtDCS improved performance compared to sham, with greater gain for participants with poorer scores at baseline.

			contralateral cheek						
Splittgerber et al., 2020 [154]	24	20 minutes; bipolar tDCS: circular 25 cm2 electrodes, 1 mA; multichannel: 3.14 cm2	Bipolar tDCS: target lDLPFC, ref: supraorbital contralateral; Multichannel: AF3, AF7, F3 - FP2, T7	atDCS, multichannel, sham (w)	online	2-back task	accuracy, RTs, EEG	2-back task pre-stimulation performance added as regressor in behavioral analyses. Correlation between pre and post stimulation EEG.	No effects at the group level. For the multichannel tDCS: low-performers improved their performance compared to sham, while high-performers worsened it. Changes at the neurophysiological level (measured within 45 minutes from the end of stimulation) were detectable for both atDCS conditions, with higher theta power for the initially low performers.
Strobach et al., 2018 [169] - Study 1	30	20 minutes; target: 5x7 cm electrodes, ref: 10X10; 1 mA	Target: rIFG (half-way between F4 and C4); ref: left supraorbital region	atDCS, sham (w)	online	Dual task	Error rates, RTs	Correlation between pre-stimulation and stimulation performance at the dual task.	Group effects: no tDCS effects over RTs, atDCS improved performance with short SOA for repeated trials (Task 1 and Task 2). Improvement was greater for participants that had a low performance at sham.
Strobach et al., 2018 [169] - Study 2	22	20 minutes; target: 5x7 cm electrodes, ref: 10X10; 1 mA		ctDCS, sham (w)	online	Dual task	Error rates, RTs	Correlation between pre-stimulation and stimulation performance at the dual task.	Group effects: no tDCS effects over RTs, ctDCS worsen accuracy in the different order condition (only at

									Task1). Inclusion of baseline led to inconclusive results (low baseline performance was not predictive of ctDCS worsening after an outlier removal).
Tseng et al., 2012 [150] - Study 2	20	15 minutes; 4x4 cm electrodes; 1.5 mA	target: rPPC (P4); ref: left cheek	atDCS, sham (w)	offline	change detection task	sensitivity indexes (d', K) – ERPs recordings	High and low performers based on their task performance in the sham condition.	No effects at the group level. AtDCS increases performance only in the low-performing group. ERPs revealed improvements in components known to be linked to attentional deployment only in the low-performing group.
Wu et al., 2020 [232]	28	20 minutes; 5 x 7 cm electrodes; 1.5 mA	target: left V5 (3 cm above and 5 cm to the left of the inion); vertex (CZ)	atDCS, sham (b)	offline	coherent motion direction identification test	Motion perception threshold	Correlation of pre-stimulation and post-stimulation performance.	Group effects: baseline motion threshold improved in all the post atDCS conditions compared to the pre-stimulation condition. Improvement was greater for participants with poorer initial performance.
Wu et al., 2021 [155]	50	20 minutes; 5x7 cm electrodes; 1.5 mA	target: primary occipital cortex (OZ); ref: vertex (CZ)	atDCS, ctDCS, sham (b)	offline	grating detection task	contrast sensitivity function	Correlation of pre-stimulation and post-stimulation performance.	No effects at the group level. AtDCS improved performance in participants with poorer initial performance and

									worsened it in participants with better initial performance.
Wu et al., 2022 [156]	56	20 minutes; 5x7 cm electrodes; 1.5 mA	target: rDLPFC (F4); ref: left supraorbital region	atDCS, ctDCS, sham (b)	offline	Go/no-go, Stroop, stop-signal task	correct rejections (go/no-go), SSRT (stop-signal task), conflict score (Stroop)	Pre-stimulation performance as covariate in the regression model.	No effects at the group level. CtDCS influences performance at the go/no-go task based on the baseline performance in two opposite directions: low performers improved, and high performers decreased their performance.

The table includes studies investigating the impact of considering individuals' baseline levels on tDCS effects. Scrolling the table from left to right, for each study, we summarized the number of participants (N); the stimulation protocols, including duration, electrodes size (or areas), current intensity; the position of the electrodes; the stimulation type ("b" indicates between participants condition, "w" within participants condition); the protocol timing (online vs. offline studies); the performed task and dependent variable included the baseline determination and study's results.

[a] (20 young and 20 older adults)

DSB = digit span backward; DSF = digit span forward; EEG = electroencephalography; ERPs = event-related potentials; HD-tDCS = high-definition tDCS; LA-RC = left anodal-right cathodal; LC-RA = left cathodal-right anodal; lDLPFC = left dorsolateral prefrontal cortex; lPPC = left posterior parietal cortex; Ospan = Operation span; PPC = posterior parietal cortex; rDLPFC = right dorsolateral prefrontal cortex; ref = reference electrode; rIFG = right inferior frontal gyrus; rPPC = right posterior parietal cortex; WM = working memory.

Table S5 summarizes studies in which task difficulty was considered in tDCS studies.

Study	N	Protocol	Electrodes' placement	Stimulation type	tDCS - task time-locking	Task	Outcome measures	Difficulty manipulation	Results
Blumberg et al., 2015 [187]	48	30 minutes; 11 cm^2; 2 mA	target: rAIPS (CP4), lDLPFC (F3); ref: contralateral upper arm	atDCS AIPS, atDCS lDLPFC, sham (b)	online	Multiple object tracking	accuracy	objects number: low (2 circles) vs. high (4 circles) load condition	Planned comparisons: accuracy improved in AIPS stimulation compared to the AIPS baseline and to the real and sham DLPFC (collapsed) only for the high load condition.
Gill et al., 2015 [194]	22[a]	20 minutes; 5x5 cm electrodes; 2 mA	target: lDLPFC (F3); ref: contralateral supraorbital region	atDCS, sham (w)	online n-back training - offline PASAT	PASAT	accuracy	n-back load: 1Back, 3Back	Performance at PASAT improved significantly only after the 3Back performance in the atDCS condition.
Hussey et al., 2015 [147]	79	30 minutes; 1.3 cm2 electrodes; 2 mA	target: lDLPFC (F3); ref: left occipital cortex (O1)	atDCS, ctDCS, sham (b)	online	Reading Task, nBack	Reading task: accuracy, nBack A'	Manipulation of task difficulty and task demands (executive control). Difficulty: long vs. short sentences, 2Back vs. 4Back. Task demands: ambiguous vs. unambiguous sentences, object vs. subject relative sentences, with-lures vs. without-lures n-Back.	AtDCS improved performance in sentences requiring higher executive control (ambiguous) than the ctDCS condition and sentences with a higher difficulty (longer) than the sham. atDCS improved nBack performance only in the high difficult condition compared to ctDCS and sham.
Kwon et al., 2015 [174]	38	15 minutes; 5 x7 electrodes; 1 mA	target: right primary motor cortex; ref:	atDCS, sham (b)	offline	Visuomotor cordination	accuracy	Movements velocity, three levels: easy, moderate, difficult	AtDCS improved accuracy compared to sham, but only at a

			supraorbital left region						moderate difficulty level.
Paladini et al., 2020 [188]	25	20 minutes; 5x7 cm electrodes; 1.5 mA	Bihemispheric tDCS: parietal cortices (P3, P4)	CL/AR, AL/CR, sham	online	Dual-task: verbal working memory task, visuospatial attention detection task	accuracy, RTs	Verbal working memory load, low cognitive load (2 consonants) vs. high (6 consonants) to be memorized	No differences between the three stimulation conditions were traceable at the low cognitive level. Conversely, an hemispheric asymmetry in RTs (shorter for right- compared to left-sided targets) was found for sham and AL/CR but was canceled by CL/AR.
Pope & Miall, 2012 [173]	60	20 minutes; 25 cm2 electrodes; 2 mA	target: right cerebellar; ref: right deltoid muscle	atDCS, ctDCS, sham (b)	offline	PASAT, PASST	accuracy, RTs	PASST is more difficult than PASAT. Both tasks were performed at an individually set difficulty level in a practice session. Participants heard numbers presented at different rates (5 trials for each speed) and performed the task at the higher velocity in which they reached 3 out of five correct answers.	PASST accuracy improved after ctDCS compared to sham and atDCS. RTs in PASST decreased after ctDCS when differences between pre and post-stimulation were computed. No differences were found in the PASAT (easier) task.
Pope et al., 2015 [149]	63	20 minutes; 25 cm2 electrodes; 2 mA	target: lDLPFC (F3); ref: right deltoid muscle	atDCS, ctDCS, sham (b)	offline	PASAT, PASST	accuracy, RTs	PASST is more difficult than PASAT. The difficulty was set for each participant	AtDCS improved all behavioral measures compared to sham and ctDCS. This effect was traceable

								by establishing an optimal individual rate in a practice session (as in Pope and Miall, 2012).	in the more demanding task (PASST), while no differences were found in the PASAT.
Pupíková et al., 2021 [191]	27	20 minutes; 5x5 cm electrodes;2 mA	anode: rMFG (MNI 44 40 - 10); cathode: rPPC (MNI = 30 -55 52)	atDCS, sham (w)	online/offline	visual object matching task (VOMT, offline), visual working memory task (VWMT, online)	accuracy, RTs	Difficulty level manipulated only for the VOMT: conventional (low) vs. unconventional (high) views.	Ceiling effects for accuracy in both tasks. AtDCS improved RTs in the higher difficulty VOMT subtask (unconventional view).
Roe et al., 2016 [172]	32	24 minutes; 5 x 7 cm electrodes; 1 mA	Bihemispheric tDCS: parietal cortices (P3, P4)	CL/AR, AL/CR, sham (w)	online	Multiple object tracking task	accuracy	Number of targets to be tracked: easy (1 target for visual field), medium (2), hard (3).	Performance worsened for the two stimulation conditions compared to sham at higher attentional load.
Sandrini et al., 2012 [193]	27	13 minutes; 5x7 cm; 1.5 ma	target: PPC bihemispheric (P3 - P4)	anodal-cathodal, cathodal-anodal, sham (b)	offline	verbal n-back task	error rates, RTs	Parametric - task difficulty*	left a-tDCS/right c-tDCS impaired RTs for 1 n-back, left anodal -left c-tDCS/right a-tDCS impaired RTs for the 2n-back
Vergallito et al., 2018 [185]	24	20 minutes	target: left and right PFC	anodal, sham (w)	online	DSB, pFT	accuracy, RTs	individualized easy, medium, high difficulties	Right atDCS decreased performance for DSB and pFT at higher difficulties. Left atDCS improved performance in the pFT for all difficulty levels.
Vergallito et al., 2020 b [189]	66	30 minutes; target: 3x3 cm, ref: 5x7	target: LIFG (F5) or LIPC (P3); ref: supraorbital	atDCS LIFG, atDCS	online	Sentence comprehension	Accuracy, RTs	Syntactic sentence complexity: coordinate, relative clause with right	For the easier sentences, LIFG stimulation increased accuracy compared to

		cm; 0.75 mA	controlateral region	LIPC, sham (b)				peripheral-embedded, relative clause with center-embedded (in increasing difficulty order)	sham conditions, while LIPC stimulation worsened performance compared to both LIFG and sham stimulations. For the two relative sentence types, no effects were traceable for LIFG stimulation, while LIPC decreased their accuracy compared to sham. tDCS did not affect RTs.
Weiss & Lavidor, 2012 [186]	29	15 minutes; target: 4x4, ref: 5x7 cm; 1.5 mA	target: rPPC (p4); ref: left supraorbital region	atDCS, ctDCS, sham (b)	online	Attentional load paradigm - flanker task	accuracy, RTs	Low-load condition: central letter presented without competing stimuli; high-load condition: central letter presented with five additional peripheral competing letters.	The well-known flanker effect was found in the low-load condition for all stimulation conditions and the high-load condition only for cathodal stimulation. This effect was interpreted as an enhancement of attentional resources due to ctDCS.

The table includes studies investigating the impact of considering individuals' baseline levels on tDCS effects. Scrolling the table from left to right, for each study, we summarized the number of participants (N); the stimulation protocols, including duration, electrodes size (or areas), current intensity; the position of the electrodes; the stimulation type ("b" indicates between participants condition, "w" within participants condition); the protocol timing (online vs. offline studies); the performed task and dependent variable included the baseline determination and study's results.

AIPS = anterior intraparietal sulcus; AL/CR = anodal left, cathodal right; ATL = anterior temporal lobe; CL/AR = cathodal left/anodal right; ctDCS = cathodal tDCS; lDLPFC = left dorsolateral prefrontal cortex; LIFG = left inferior frontal gyrus; LIPC = left inferior parietal cortex; PASAT = paced auditory serial addition task; PASST = paced auditory serial subtraction task; PFC = prefrontal cortex; pFT = paced finger tapping; PPC = posterior parietal cortex; rAIPS = right anterior intraparietal sulcus; rMFG = right middle frontal gyrus; rPPC = right posterior parietal cortex.

[a] Eleven participants for each experiment (Experiment 1 and Experiment 2), analyses were run by combining the two experiments.

[b] the database includes a subset of participants analyzed in Giustolisi et al. (2018).

Table S6 summarizes the studies in which current intensity and electrode montage were manipulated to investigate tDCS effects. Electrode configuration is reported using EEG 10-20 references; target electrode (mainly anode) is reported before the reference electrode (mainly the cathode) (i.e., target electrode + reference electrode).

Current intensity and electrodes montage									
**Study**	**Factor**	**Study Design**	**N**	**Stimulation protocol**	**Electrodes' montage (target+ref. electrode) and dimension**	**Target area**	**Task**	**Outcome Measures**	**Results**
Batsikadze et al., 2013 [211]	Current intensity, electrode position	Real tDCS study	14 healthy subjects (9 females; mean age 25.8 ± 3.7 years)	Single sessions of 20 min offline a-tDCS, c-tDCS and sham at 1 mA and 2 mA	Electrode montage: left M1 + right SO. Electrode size: 35 cm^2.	motor cortex	-	MEPs, SICI, ICF	tDCS intensity enhancement might shift the direction of excitability. 1 mA c-tDCS reduced MEPs amplitudes and shifted SICI and ICF towards inhibition, while at 2 mA both a- and c- tDCS increased MEPs amplitude.
Dmochowski et al., 2013 [209]	Electrode position	Real tDCS study	8 aphasic patients were included in a pilot study	Single session of a-HD-tDCS at 2 mA	Conventional vs. optimized montage with 4 active electrodes (2 anodes and 2 cathodes). The target area was determined for each participant from fMRI data acquired during an overt picture-	perilesional cortex (frontal, parietal, temporal cortex)	a self-administered computerized treatment in which an audio stimulus is paired with a picture	Electric field distribution	EF strengths in the targeted cortex increase up to 63% when optimizing the electrode configuration with fMRI/MRI to define a target with respect to the conventional approach.

					naming recall task. Electrode size: 6mm.				
Evans et al., 2020 [206]	Current intensity	Simulation study: realistic head models derived from structural MRI of 50 healthy adults (aged 22-35; 23 males, 27 females) taken from the Human Connectome Project	-	Single session of a-tDCS at 1 mA, 2 mA and individualized dose	Electrode montage: CP5 + FC1. Electrode size: disc electrodes of 6 mm radius and 2 mm height.	motor cortex	-	Electric field distribution	EF intensity in left M1 varied by more than 100% across individuals throughout the brain with a fixed dose (1 mA; 2 mA). Individualized dose-control ensured the same EF intensity at the cortical target site.
Hanley et al., 2020 [101]	Current intensity, aging	Real tDCS study	40 old adults (20 females; mean age 67.05 ± 5.21 y.o.)	Three offline sessions of 10 and 20 min a-tDCS and sham at 1.5 mA	Electrode montage: F3 + F4. Electrode size: 25 cm^2.	DLPFC	a complex task-switching paradigm	RTs	10 minutes of a-tDCS significantly improved task-switching speed from baseline, contrary to the sham-control and 20 min variant in an aging population.
Hoy et al., 2013 [214]	Current intensity	Real tDCS study	18 healthy participants (11 females; mean age 24.71 ± 6.97 years).	Three 20 min offline sessions of a-tDCS and sham at 1 mA and 2 mA	Electrode montage: F3 + right SO. Electrode size: 35 cm^2.	DLPFC	working memory task (n-back task)	RTs; accuracy; EEG frontal activity	Increasing the tDCS dose does not induce greater or longer-lasting behavioral or neurophysiological effects in healthy controls. The greatest improvement was

									seen following 1 mA stimulation.
Kashyap et al., 2021 [215]	Current intensity	Simulation study: realistic head models derived from structural MRI of 90 healthy adults (aged 22-35; 45 females) taken from Cambridge Centre for Ageing and Neuroscience	-	Three sessions of a-tDCS at 1 mA, 2 mA, and 3 mA	Electrode montage: F3 + right SO. Electrode size: 25 cm^2.	DLPFC	-	Electric field distribution	The non-linear relationship between the injected tDCS current and the distribution of CD in the target area is predominant in older adults with a decrease in focality. The decline is stronger in males. A higher current dose at an older age can enhance the focality of stimulation. The recommended dose–target determination index dose should be prioritized based on the age (>40 years) and sex (especially for males) of an individual.
Kidgell et al., 2013 [220]	Current intensity	Real tDCS study	14 healthy participants (6 females; range: 22–45 years)	Three sessions of 10 min a-tDCS at 0.8 mA, 1 mA and 1.2 mA	Electrode montage: C3 + contralateral SO. Electrode size: 25 cm^2.	motor cortex	-	MEPs, SICI	No differences were found between the three intensities of a-tDCS on modulating cortical excitability or SICI. Cortical excitability increased, and SICI

									was reduced following a-tDCS.
Laakso et al., 2019 [208]	Current intensity	Real tDCS study	28 healthy adults (7 females; mean age=27 ± 6 years)	Two 20 min offline sessions of a-tDCS and sham at 1 mA	Electrode montage: right M1 + contralateral SO. Electrode size: 25 cm^2.	motor cortex	-	MEPs	Subjects with the weakest and strongest EF produce opposite changes in excitability, as shown by MEPs. The effective EF component was in the direction normal to the cortical surface. EF dosimetry could help control the neuroplastic effects of tDCS.
Nikolin et al., 2018 [221]	Current intensity	Real tDCS study	100 healthy subjects (9 females; mean age: 22.9 ± 4.3 years)	Experiment 1 (40 subjects): 15 min offline a-tDCS at 1 mA and sham (b). Experiment 2 (60 subjects): 15 min offline a-tDCS at 2 mA or "off condition" or sham (b).	Electrode montage: F3 + F4. Electrode size: 16 cm^2.	DLPFC	Working memory task.	RTs, accuracy, and event-related EEG component (P3).	1 mA, 2 mA and sham conditions had biological effects measured with P3 EEG component, with the largest effect size for 1 mA stimulation. Sham stimulation previously considered inactive may alter neuronal function. Working memory performance was not significantly altered by tDCS, regardless of dose.
Teo et al., 2011 [212]	Current intensity	Real tDCS study	14 healthy participants	Three 20 min online sessions of a-	Electrode montage: F3 + right SO.	DLPFC	working memory task	RTs; accuracy	There were no significant improvements in

				tDCS and sham at 1 mA and 2 mA	Electrode size: 35 cm^2.		(3n-back task)		participants' accuracy, but a significant interaction was found for current strength and time for accurate reaction time.
Wiethoff et al., 2014 [27]	Current intensity	Real tDCS study	53 healthy subjects (33 females; mean age 26.83 ± 8.97 years)	Single session of 10 min offline a-tDCS at 2 mA	Electrode montage: left M1 + right SO. Electrode size: 35 cm^2.	motor cortex	-	MEPs	50% of individuals had only a minor/no response to tDCS, whereas the remainder had a facilitatory effect on both forms of stimulation. Correlation between the latency difference of MEPs and the response to a- but not c- tDCS.
Workman et al., 2019 [216]	Current intensity	Real tDCS study	34 healthy young adults (22 females; mean age 24 ± 3.6 years)	Two 20 min online sessions of a-tDCS and sham at 4 mA	Electrode montage: C3 + contralateral SO. Electrode size: 35 cm^2.	motor cortex	strength and fatigue test	Sensation tolerability and fatigue	4 mA tDCS is well tolerated by young, healthy subjects and increases left knee flexor fatigability.
Workman et al., 2020 [217]	Current intensity	Real tDCS study	34 healthy young adults (22 females; mean age 24 ± 3.6 years)	Three 20 min online sessions of a-tDCS and sham at 2 mA and 4 mA	Electrode montage: C3 + contralateral SO. Electrode size: 35 cm^2.	motor cortex	strength and fatigue test	Sensation tolerability and fatigue	Males and females report different sensation severities from 2 mA and 4 mA tDCS. Females report higher severities with increasing intensity (sham < 2 mA < 4 mA). Men reported similar severities in

									all stimulation conditions.

The table includes the studies in which current intensity and electrodes montage were manipulated to investigate tDCS effects. Scrolling the table from left to right, for each study, we summarized the studies features and design (real or simulated), the number of participants (N), stimulation protocols including duration and current intensity, electrodes position, the targeted brain area, the task performed by participants, the outcome measure and results. Electrode configuration is reported using EEG 10-20 references; target electrode (mainly anode) is reported before the reference electrode (mainly the cathode) (i.e., target electrode + reference electrode).

ADM= abductor digit minimi; a-tDCS= anodal tDCS; (b) = between; CD= current density; CSE= cortico-spinal excitability; CSF= cerebrospinal fluid; c-tDCS= cathodal tDCS; DLPFC= dorsolateral prefrontal cortex; EF= electric field; FCR= flexor carpi radialis; FDI= first dorsal interosseous;GM= gray matter; HD-tDCS= high definition tDCS; ICF= short latency intracortical facilitation; MEPs= motor evoked potentials; MRS= magnetic resonance spectroscopy; M1= primary motor cortex; PFC= prefrontal cortex; RTs= reaction times; SICI= short latency intracortical inhibition; SO= supraorbital area; WM= white matter.

Table S7 summarizes in-vivo human studies in which intracranial cortical recording was used to investigate the induced electrical fields.

Study	N	Participants' diagnosis	Stimulation Type	Protocol	Stimulated regions	Implanted electrodes	Results
Chhatbar et al., 2018 [224]	Three participants (males: 60, 71, 77 y.o.)	Movements disorders (essential tremors, Parkinson's disease)	tDCS	5x7 cm electrodes; 2 and 4 mA; 3 minutes	2 montages: bitemporal (anode over the left temple, cathode over the right); occipitofrontal (anode over the occipital bone, cathode over the forehead)	Deep brain stimulation (DBS) electrodes, subthalamic nuclei	tDCS produces an EF inside the brain in a dose-dependent and montage-specific way.
Huang et al., 2017 [226]	Ten participants	Epilepsy	tACS	2 x 2 cm; 0.25-2 mA	mid-forehead (Fpz), occiput (Oz)	Implanted subdural electrodes	EFs were predicted with several computational current-flows models. Estimated EFs correlated with the recorded values both in cortical and deep electrodes (r = .86 and r = .88) respectively.
Louviot et al., 2021 [222]	Eight participants (4 males, age: 30 ± 11 y.o.)	Drug-resistant epilepsy	tACS	HD electrodes (4.52 cm^2); seven frequencies (one patients); two intensities (six patients)	15 montages (one patient)	Stereoelectroencephalographic electrodes	tACS can induce EF in deep brain structures. EF magnitude correlates to stimulation intensity and is influenced by electrodes montage.
Opitz et al., 2016 [225]	Two participants (male 29 y.o., female 35 y.o.) and two nonhuman primates	Refractory epilepsy	tACS	25 cm^2; 1 mA; 1 Hz; 2 minutes	Bilateral temporal areas (left and right temples)	One patient had bilateral stereotactic electrodes; the other one had left subdural grid, strip, and depth electrodes.	The results highlight that maximum EF in humans reaches up to 0.5 mV/mm for 1 mA stimulation, in line with modeling previsions. Inter-individual differences in the EF strength were found.

Ruhnau et al., 2018 [223]	Single case (64 y.o. woman)	Movement disorders	tACS	5x7 cm; 1 mA; 10 vs. 130 Hz	Bilateral temporal areas (T7-78)	subcortical structures (ventromedial nucleus and globus pallidus internus)	The modeling of the current flow is accurate. A significant amount of current reach even deep brain structures.

The table describes the studies investigating in-vivo induced electric fields in the human brain. Participants' number and age, diagnosis, stimulation type and protocol, the stimulated regions, implanted electrodes position, and results are summarized.
